# Effect of Types of Microparticles on Vibration Reducibility of Cementitious Composites

**DOI:** 10.3390/ma15144821

**Published:** 2022-07-11

**Authors:** Siyu Wu, Sungwoo Park, Sukhoon Pyo

**Affiliations:** Department of Urban and Environmental Engineering, Ulsan National Institute of Science and Technology (UNIST), Ulsan 44919, Korea; wsy86023@unist.ac.kr

**Keywords:** hollow microsphere glass, cenosphere, graphite flakes, damping ratio, strength, cementitious composites

## Abstract

The vibration-reducing ability of construction materials is generally described by the damping ratio of the materials. Previously, many studies on the damping ratio of concrete have been done, such as the addition of rubber, polymer, fiber, and recycled aggregates in the concrete. However, the application of these materials in construction is limited due to their drawbacks. This paper investigated the effect of the replacement ratio and the size of the hollow glass microspheres (HGM), cenospheres (CS), and graphite flakes (GF) on the damping ratio of mortar. Furthermore, rubber particles (RP), aluminum powder (AP), and natural fiber (NF) were investigated to find if they have a combination effect with HGM. The half-power bandwidth method was conducted to obtain the damping ratio at 28 days of curing, and the compressive and flexural strength tests were also conducted to study the mechanical properties of mortar that contained HGM, CS, and GF. The results show that increases in the size of HGM and the replacement ratio of sand with HGM lead to an increase in the damping ratio. Moreover, RP and NF do not provide a combination effect with HGM on the damping ratio, whereas the application of AP results in a drastic compressive strength decrease even with an increase in damping ratio when incorporated with HGM. Besides, an increase in the replacement percentage of CS also leads to an improvement in the damping ratio, and a smaller size and higher replacement ratio of GFs can improve the damping ratio compared to other additives. As a result, CS and GF are more effective than HGM. 50% replacement ratio of CS slightly reduced the compressive strength by 6.4 MPa while improving the damping ratio by 15%, and 10% replacement ratio of samller GF can enhance the flexural strength by over 4.55% while increasing the damping ratio by 20.83%.

## 1. Introduction

Subway and railway systems are major transport systems in metropolitan cities, designed to deal with the increasing demand for public transportation. However, it is hard to avoid building the viaducts of subway or railway, which run through a large swath of residential areas, and when the trains pass on the viaduct, the vibration and the structure-borne noise inevitably transmit through the concrete viaducts and radiate to the nearby residents [1,2], which has great negative influences on the occupant comfort and health [3,4]. To reduce the vibration from the train passage, engineers applied dampers to the viaduct, installed the floating track [5], and attached the vibration isolating layers on the track slab [6]. However, construction materials are the main vibration transmission medium, so improving the vibration reduction capacity of construction materials is a more effective way.

In the construction material field, many researchers used different kinds of additives to improve the damping capacity of building materials based on cement-matrix composites. However, the additives used in previous studies caused some problems due to their drawbacks. Recently, recycled tire rubbers have been used to replace part of the aggregates on concrete to solve the serious environmental problem [7,8]. Although rubbers can improve the damping ratio of concrete due to the viscoelastic property, the damping ratio of concrete with rubber decreases with ages because the rubber will lose its viscoelasticity after several vibration cycles [9]. Besides, to reduce the environmental impact, energy consumption and greenhouse gas emissions, some researchers reutilized solid waste from the concrete industry as recycled aggregates (RA) to make concrete [10,11]. It has been proved that 100% replacement of coarse aggregate by recycled aggregates could enhance the damping property of the concrete, but improvement of damping capacity is less than 10%, although the replacement of recycled aggregates reaches 100% [10,12]. The addition of air-entraining agents in concrete can also improve the damping ratio due to the increased porosity of concrete, and the pores inside work as cushioning bags to absorb vibration, but high porosity negatively affects the mechanical properties of concrete [13]. Moreover, different fibers such as synthetic fibers, nanofibers, and natural fibers were studied to add to concrete to improve energy dissipation [14,15,16]. It was found that the high volume fraction could introduce a new interfacial transition zone (ITZ), which can increase the internal friction so that energy dissipation can be improved. However, fiber volume content is limited for normal concrete, and it was proved that a low volume fraction of fibers such as polyolefin fiber, steel fiber, and coir fiber had no significant effect on the damping ratio of concrete, and the damping ratio of fiber reinforced concrete decreased as time went by [17]. It has been found that carbon nanotubes could improve the damping ratio of cement-based composites [18], but it is not easy to uniformly disperse in concrete due to its agglomeration effect [19]. Therefore, this study aims to utilize other types of materials to compensate for the drawbacks of the materials as mentioned above while improving the damping ratio.

Unlike previous research, this study investigated the utilization of hollow glass microspheres, cenosphere, and graphite flakes in the cement mortar to improve the damping ratio while trying to avoid the drawbacks of other materials as mentioned above. Although previous studies applied hollow glass microspheres (HGM) and cenospheres (CS) to develop lightweight concrete [20,21,22], and graphite flakes (GF) were used for controlling the conductivity of concrete [23], there is no existing study focusing on evaluating the damping ratio of mortar that contained these materials. Compared with rubber particles (RP), RA, and fibers, HGM has several advantages, such as high thermal resistance, reducing shrinkage, and improving the workability of mortar [24]. Especially, HGM particles have hollow structures inside filled with air, which can lower the vibration transmission speed to increase the energy dissipation of concrete because vibration transmission speed is slower in the air than in solid structures. Likewise, CS particles also have hollow structures inside filled with air or inert gas, and CS is a kind of byproduct from coal combustion at thermal power plants, so the utilization of CS in concrete as filler helps alleviate the environmental problem [25]. Different from HGM and CS, GF is highly crystalline and consists of multi-layers of graphite and Van der Waals force that is a weak bond among each layer, which allows each layer to slide one past another resulting in a preferential shear direction of deformation [26]. It is known that this microstructure can improve the shear modulus of asphalt. Moreover, the addition of GF in asphalt can improve the deformation resistance of asphalt [27], which means that it can improve the stiffness that is a critical factor for vibration reduction [28]. Therefore, this study aims to increase the vibration-reducing ability without sacrificing or slightly decreasing the compressive and flexural strengths of mortar by using these three additives.

In this study, cement mortar specimens were prepared by replacing sand with HGM, CS, and GF with various volume fractions, and the damping ratio was evaluated through a longitudinal impact test and half-power bandwidth method with beam specimens. The effectiveness of the volume fractions of HGM, CS, and GF, and the size of HGM and GF for improving the damping ratio was studied. Also, some specimens were prepared with HGM and other possible damping ratio improving materials such as RP, air-entraining agent, and natural fiber (NF) to investigate if any material has a combination effect with HGM on the damping ratio. Furthermore, the compressive and flexural strengths were measured through the compression test of cubic specimens and the three-point bending test with the beam specimens after the damping ratio test. The thermogravimetric analysis (TGA) was conducted to find more reasons for mechanical strength and damping ratio differences.

## 2. Materials and Test Methods

### 2.1. Materials

**Figure 1:**
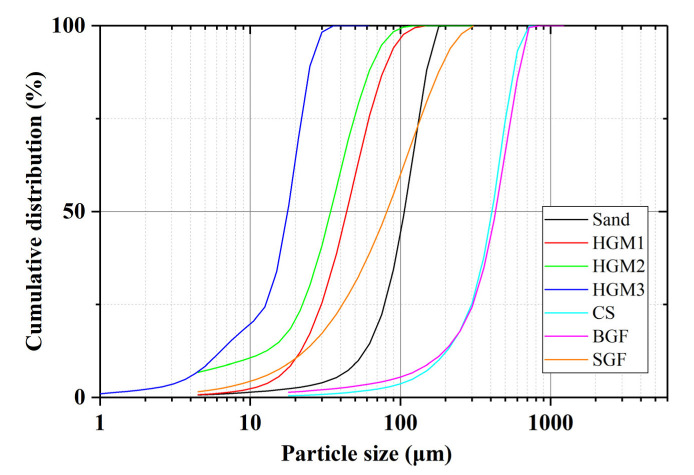
Particle size distribution of HGMs, CS, GFs, and sand.

The cement used in mortar specimens was ordinary Portland cement (CEM I 52.5). The chemical composition of raw materials, including CS and HGM, is shown in Table 1. In this study, RP and NF are provided by Hangil RMB and Soo Industry Co., respectively. The NF used in this study is a kind of kenaf fiber, and its tensile strength and Young’s modulus are 930 MPa and 53 GPa [29], respectively. This study has used three different HGMs, to investigate the impact of HGM particle size on the damping ratio supplied by 3M^TM^, and the three HGMs are labeled as HGM1, HGM2, and HGM3, respectively. CS is supplied by Seokyung Cmt, which is the fly ash lighter than water and produced during coal combustion. The diameter range of CS particles is from 50 μm to 800 μm. The GF was obtained from the Merck KGaA, and two different GFs were used in this study to investigate the effect of size on the damping ratio; one has a particle size less than 150 μm, and the other one has a particle size larger than 300 μm, and they labeled as SGF and BGF, respectively. The fine aggregate used was river sand with a specific gravity of 2.60. The physical properties of additives are shown in Table 2, and Figure 1 shows the particle size distribution of sand, HGMs, CS, and GFs. It can be found that the crushing strength of HGM increases with the increase of density and decrease of HGM particles. This is because the wall thickness of HGM particles determines the crushing strength, which increases with a decrease in the particle size [30].

### 2.2. Mixture Proportion and Specimen Details

The mixture proportions are shown in Table 3. Seventeen different mixtures were prepared. Take 30R25HGM1 as an example, 30R means replacing 30% volume fraction of sand with RP and 25HGM1 means that 25% volume fraction of sand was replaced by HGM1. However, only for 0.05AP and 0.05AP25HGM1 specimens, the AP addition content was based on the weight of cement because it was used as an air-entraining agent with a very small dosage. The water to cement ratio is 0.45 for all specimens.

Three 50 mm cubic blocks and three 40 × 40 × 160 mm prisms were cast for each mixture. The prisms were used for damping ratio tests and flexural strength tests, and cubic blocks were used for compressive strength tests at 28 days. All the specimens were kept in the molds for 24 h under laboratory conditions. Then the specimens were removed from the molds and cured under the water curing condition for 27 days.

### 2.3. Test Methods

#### 2.3.1. Compressive Strength and Flexural Strength Tests

Compressive and flexural strength of specimens were tested at 28 days according to ASTM C109 [34] and C293 [35], respectively. The compressive strength was measured under a loading rate of 1.5 kN/s, and the compressive strength test was conducted for the three cubic blocks. The average value of three testing results was obtained as the final compressive strength for each mixture. On the other hand, the flexural strength was measured with a three-point bending test under a loading rate of 355.6 N/min after damping ratio test. The final flexural strength was also determined by the average value of three beam specimens for each mixture.

#### 2.3.2. Damping Ratio Test

**Figure 2:**
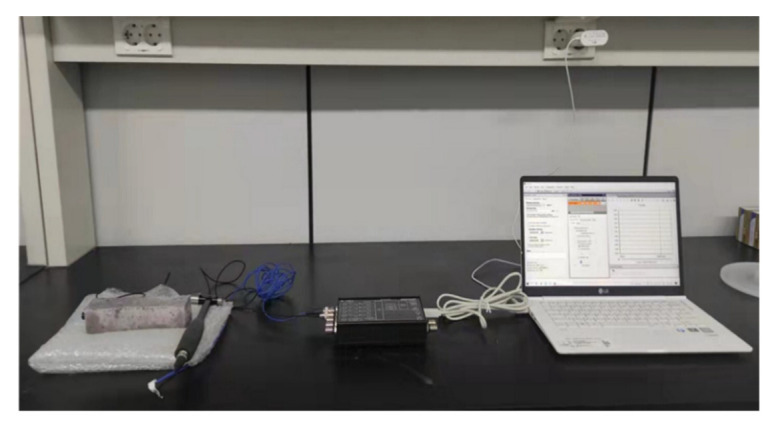
A picture of the damping ratio test setup.

The damping ratio of mortar was evaluated by the vibration of a free-free beam according to ASTM C215 [36]. An acceleration sensor Type KS95C.10 (IDS Innomic GmbH, Germany), data acquisition, and analysis software (QuickDAQ 3.7.0.47) were used in this test. Figure 2 shows the setup of the damping ratio test, a sensor was attached to the surface of one end of the specimen and a standard impact hammer was used to apply the impact force to the surface of the other end of the specimen in the longitudinal direction [37]. All specimens are the same prism specimens for the flexural strength test. The damping ratio based on the half-power bandwidth method is expressed as follows:

(1) $$\;\xi =\frac{f_{1}-f_{2}}{2f_{0}}$$

where, *ξ* is the damping ratio of specimen, *f*_0_ is the resonance frequency of specimen, *f*_1_, *f*_2_ are the frequencies corresponding to an amplitude of $f_{0}/\sqrt{2}$. The value of these parameters can be provided by the frequency response function (FRF) graph from analysis software [38].

#### 2.3.3. Thermogravimetric Analysis for HGM and CS Specimens

In this study, thermogravimetric analysis was performed with a TGA system Q500 (TA Instrument, New Castle, DE, USA) with the nitrogen carrier gas and at a heating rate of 10 °C/min from 30 to 900 °C. All the HGM and CS specimens were ground into powder and processed by a solvent exchange method for preparing the TGA test [39,40].

## 3. Results and Discussion

### 3.1. Damping Ratio

**Figure 3:**
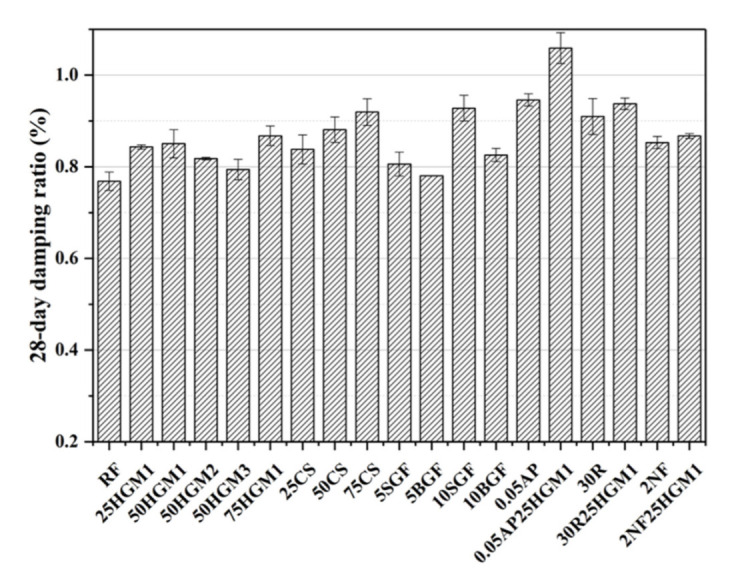
28-day damping ratio of specimens.

The damping ratio test results are shown in Figure 3. All the specimens have a higher damping ratio than the RF specimen. The improvement of the damping ratio of specimens with different additives is different. To find out how the size of HGM affects the damping ratio, HGM1, HGM2, and HGM3 were separately added in the mortar. It can be found that the damping ratio decreased with the increase of HGM strength and the decrease of HGM size at a 50% HGM replacement ratio. This is because the wall thickness of HGM particles increases when strength increases and size decreases. In other words, less air was trapped in the hollow structure of smaller HGM particles, so there were fewer voids in the mortar, and the energy dissipation decreased while the size of HGM decreased. This is because the damping ratio increases with the increase of the number of voids in specimens [41], and the vibration more easily transmits through the smaller wall thickness of HGM particles. Therefore, the larger size HGM is better for damping ratio improvement, and HGM1 is the best option among HGMs in this study in terms of damping capacity. 

Moreover, the damping ratio increased with the increase of HGM1 content compared to the RF specimen because air or gas was trapped in the hollow structure of HGM particles, and more HGM1 means more voids in mortar, and the transmission speed of vibration was lower in the air than that in the solid structure [42], and increasing the voids can improve the damping ratio [41]. Thus, the energy dissipation can be improved, but the high replacement ratio of HGM1 (50HGM1 and 75HGM1) only slightly increases the damping ratio compared to 25HGM1, and the possible reason will be discussed in Section 3.4.

**Figure 4:**
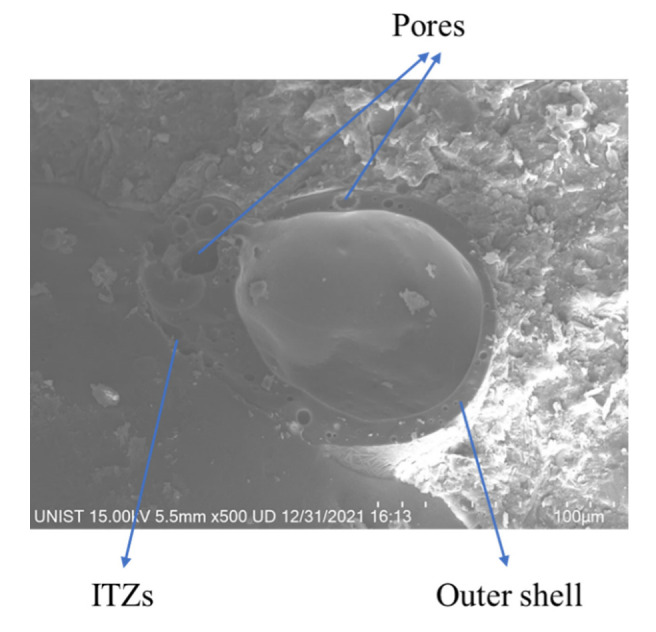
An SEM (Hitachi High-Technologies Co., Tokyo, Japan) image of cenosphere in the cement matrix.

Similarly, the damping ratio of the specimen with CS increases with the increase in the replacement ratio of CS due to the trapped air or inert gas in the hollow structure of CS particles. Compared to the RF specimen, the damping ratio of 25CS, 50CS, and 75CS increased by 9.11%, 14.7%, and 19.79%, respectively. Moreover, at the 25% replacement ratio, the damping ratio of 25HGM1 and 25CS is almost identical, but when the replacement ratio reaches 50% and 75%, the damping ratio of 50CS and 75CS is 3.53% and 6% higher than 50HGM1 and 75HGM1, respectively, because the porous shell of the CS surface, as shown in Figure 4, provides weak ITZs [43], and this can provide the sliding effect between CS particle and cement matrix during vibration; thus, this can also contribute to energy dissipation in the mortar [10,17]. Therefore, CS can improve the damping ratio more than HGM1 at a higher replacement ratio.

**Figure 5:**
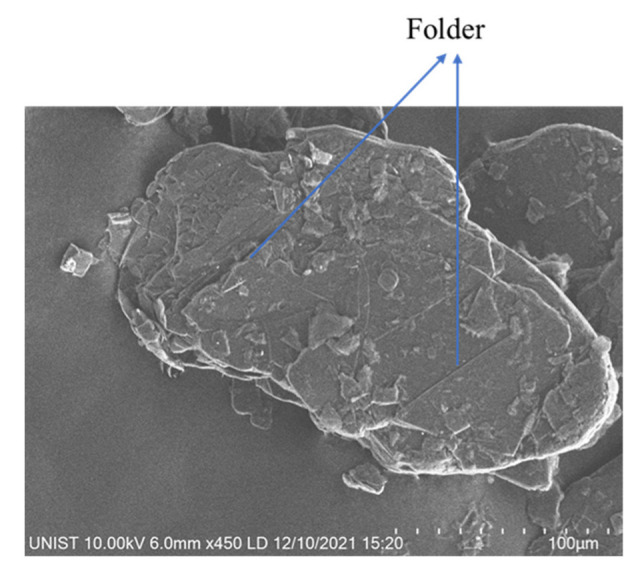
A SEM image of SGF.

On the other hand, a small amount of GFs can improve the damping ratio. Only a 5% volume replacement ratio of GFs can improve the damping ratio by 4.95% (5SGF) and 1.56% (5BGF). Moreover, with the increase in the replacement ratio of GFs, the damping ratio increased by 20.83% (10SGF) and 7.55% (10BGF) compared to the RF specimen. The results show that the SGF has a better effect on the damping ratio than BGF because the SGF particle has a larger specific surface area than BGF. Besides, the folder on the GFs surface can also provide the internal friction between GFs particles and cement matrix, as shown in Figure 5. And Van der Waals force between layers of GFs particles is weak, which provides the sliding between layers in microstructure during vibration so that damping ratio can be enhanced [26]. Furthermore, it has been found that GFs can improve deformation resistance [27]. Therefore, it can be concluded that GFs were more effective than HGM and CS in damping ratio improvement in terms of the replacement ratio.

For investigating the possible materials that have a combination effect with HGM1 on the damping ratio, AP, RP, and NF were added separately with HGM1. It can be found that when adding HGM1 with RP or NF in mortar, the damping ratio is slightly higher than the specimen that only contained RP or NF. However, the damping ratio of the specimen that contains both AP and HGM1 increases to 1.06%, which was 38.02% and 11.95% higher than the RF specimen, and the specimen only contained AP, respectively. Therefore, only the AP had a combination effect with HGM1 on the damping ratio because both AP and HGM increased the voids of specimens, but the compressive strength of 0.05AP25HGM1 drastically decreased compared to the RF specimen. This will be discussed in Section 3.2. Therefore, it is difficult to increase the damping ratio with HGM and another damping-improving material without decreasing or slightly decreasing compressive strength.

### 3.2. Compressive Strength

**Figure 6:**
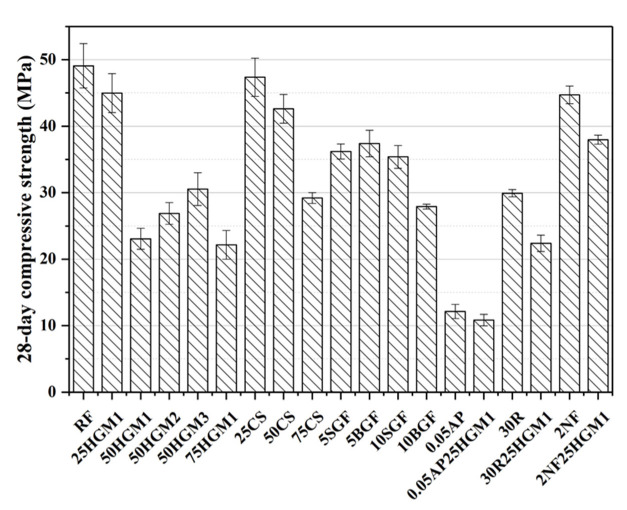
28-day compressive strength of specimens.

The compressive strengths of all specimens are shown in Figure 6. All additives used in this study decreased the compressive strength compared to the RF specimen, but the decrement of compressive strength differed with different additives. For the HGM specimens, when the HGM1 replaced the 25% volume fraction of sand, the compressive strength decreased by 8.39% compared to the RF specimen. With the increase of the HGM replacement ratio, the compressive strength significantly decreased over 50% after the replacement ratio reached 50% It can be found that a 25% replacement ratio is the critical value for HGM1 in this study. Although HGM2 and HGM3 have much higher crushing strength than HGM1, as can be seen in Table 2, the compressive strength of the 50HGM2 and 50HGM3 specimens was 16.51% and 32.42% higher than 50HGM1, respectively, and the compressive strength of these three specimens was still over 37% lower than the RF specimen. The decrease in compressive strength can be attributed to the low crushing strength of HGM. Given that HGM is non-reactive under normal curing conditions [31,32] and cannot contribute to strength development, which will be discussed in Section 3.4.

On the other hand, CS also decreased the compressive strength, and the CS with a replacement ratio of 25% and 50% only decreased the compressive strength by 3.52% and 13.20%, respectively, compared to the RF specimen. Moreover, the compressive strength drastically decreased by 40.52% at 75% replacement ratio of CS. However, the 50CS and 75CS specimens had higher compressive strength than HGM1 specimens with the same replacement ratio due to the higher crushing strength of CS than that of HGM1, as shown in Table 2. The decrease in compressive strength is due to the large volume fraction of CS that causes many weak interfacial transition zone (ITZs) [43] between the CS particle and mortar matrix due to the pores in the outer shell of CS particle [44], as shown in Figure 4, which led to the drop in compressive strength. 

GF specimens showed different compressive strength trends compared with other types of microparticles. The compressive strength of 5SGF, 5BGF, and 10SGF decreased by about 25%, whereas the compressive strength of 10BGF decreased by 43.12%, compared to the RF specimen. Besides, with the increase of the replacement ratio of BGF, the compressive strength decreased while the compressive strength of the specimen with SGF did not change. This is because the flake shape is also weak in compression [45]. The BGF led to lower compressive strength due to the larger flake shape. Although the SGF with a 10% volume replacement ratio did not decrease the compressive strength compared to the specimen with a 5% replacement ratio of SGF, it can significantly enhance the energy dissipation of mortar compared to other additives, as discussed in Section 3.1.

The combination of HGM1 and RP, AP, or NF decreased compressive strength compared to the RF specimen. Especially, the compressive strength of (0.05AP and 0.05AP25HGM1) decreased by 75.29% and 77.91% after 28 days of curing, respectively. This is because AP significantly increased the porosity of specimens. Besides, the specimens containing AP, RP, or NF with HGM1 also had lower strength than the specimen containing only AP, RP, or NF, respectively. This is caused by the extremely low crushing strength of HGM1, as shown in Table 2.

### 3.3. Flexural Strength

**Figure 7:**
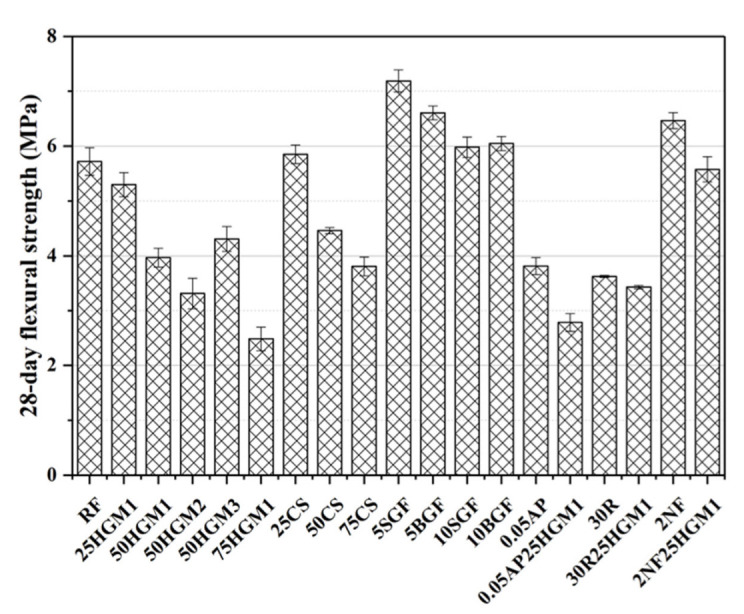
28-day flexural strength of specimens.

Figure 7 also shows the flexural strength of specimens. Under the flexural tension stress, the test specimens showed a similar trend to the behavior under compressive stress, except the specimens contained GF. For the specimens with HGM, the flexural strength decreased with the increase of the HGM content, and the specimens with HGM3 had the highest flexural strength among HGM, but its flexural strength was still 24.65% lower than the RF specimen, and this is due to the low crushing strength of HGMs. The specimens that contained the CS had a similar trend as HGM specimens, and the flexural strength also decreased with the increase in the replacement ratio of CS. The flexural strength of the 25CS was similar to the RF specimen with a low replacement ratio. However, the flexural strength decreased at a higher replacement ratio of CS.

Moreover, the flexural strength of specimens that contain AP or RP are about 33% lower than the RF specimen, whereas the 2F25HGM1 specimen slightly decreased the flexural strength by 2.45% due to the low strength of HGM1. However, the fiber-reinforced specimens provide comparable flexural strength; the 2NF specimen increased the flexural strength by 13.11%. Besides, all specimens with GF increased the flexural strength by 25.70% (5SGF), 15.56% (5BGF), 4.55% (10SGF), and 5.77% (10BGF), respectively, because GF played a role as reinforcement in mortar, similar functions as in particulate composites [46], and the reinforcement effect of GF in flexural strength was dominant. In other words, the addition of GF can enhance the deformation resistance of concrete; thus, the flexural strength can be improved. Furthermore, the flexural strength decreased with the increase of GF content due to the lubricant effects of more GF particles [27], and this is because GF is also used as a kind of solid lubricant [47], so a low dosage of GF is promising to improve the flexural strength of mortar while improving damping ratio.

### 3.4. TGA Results of HGM and CS Specimens

**Figure 8:**
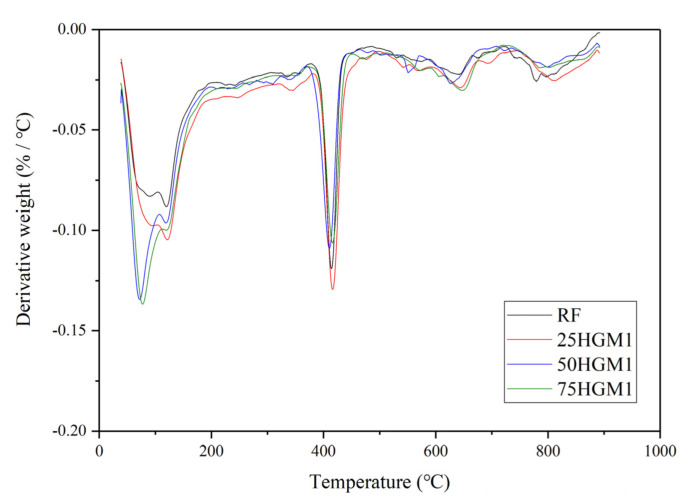
TGA results of HGM1 specimens.

**Figure 9:**
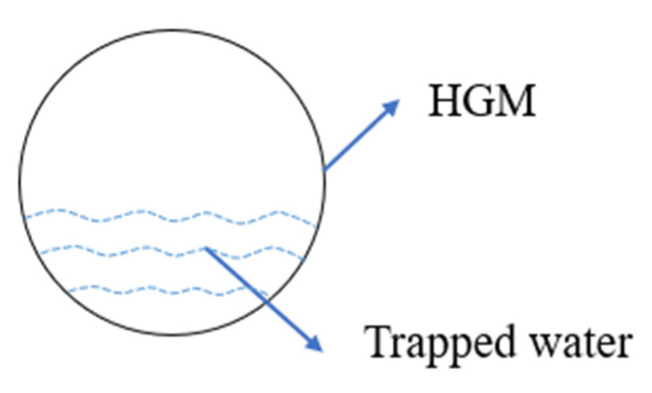
Trapped water in the HGM particles.

**Figure 10:**
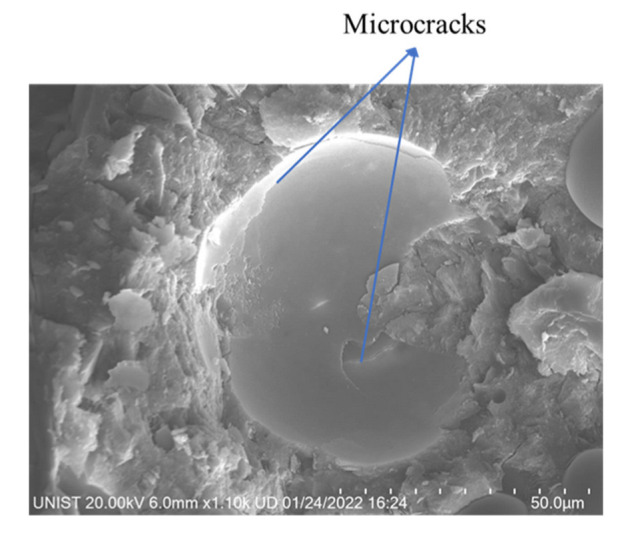
Microcracks on the HGM1 particle surface in the cement matrix.

Figure 8 shows the TGA results of specimens with HGM1 at different replacement ratios. The weight loss in TGA was normalized by cement weight because the mass ratio of cement was different for each specimen due to the various densities of HGMs based on sand volume replacement. For HGM specimens, the first peak between 60 °C and 105 °C is related to the evaporation of bound water. It can be found that the remained water content increased with the increase in the replacement ratio of HGM1. Figure 9 shows the possible way that water is trapped in the hollow structure because of microcracks on the HGM particle surface, as shown in Figure 10. When the replacement ratio of HGM1 increased, the water penetrated into the hollow structure through the microcracks after 28 days of curing. The second peaks between 120 °C and 150 °C and the third peaks between 400 °C and 500 °C of the three HGM1 specimens are very close to the RF specimen, which means HGM1 did not participate in hydration and pozzolanic reaction. This was also corresponding to the compressive strength results. Moreover, the energy dissipation was affected when the trapped water increased. Although the damping ratio increased with the increase in the replacement percentage of HGM1, the increment was only 3.21% between 75HGM1 and 25HGM1 because the trapped water filled in the hollow structures. It eliminated the effect of HGM1 particles on the damping ratio because vibration transmission speed is higher in water than in air.

**Figure 11:**
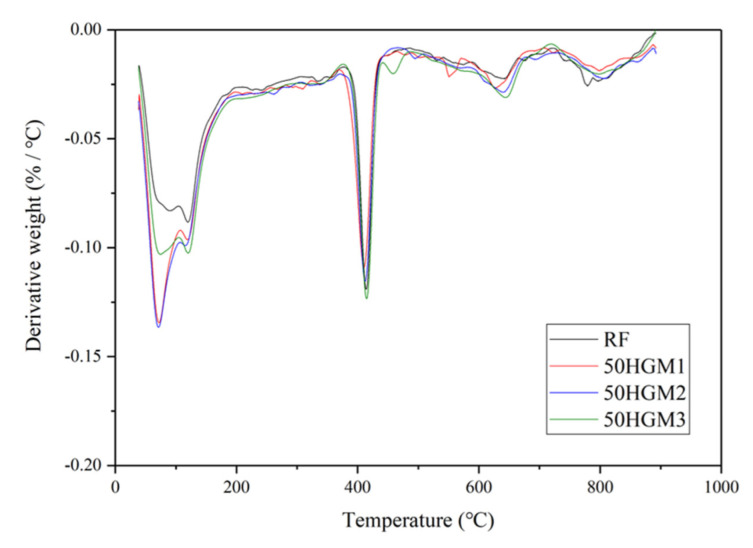
TGA results for specimens at a 50% replacement ratio with different HGMs.

Figure 11 shows the TGA results for specimens with different HGMs at a 50% replacement ratio. The specimens contained larger HGM so that more trapped water in specimens. It can be found that the HGM3 specimen showed a lower first peak than HGM1 and HGM2 specimens. This is due to the larger wall thickness and smaller size of HGM3 than HGM1 and HGM2, and less water can penetrate into the hollow structure. The first peaks of HGM1 and HGM2 specimens are close because the difference in size between these two HGMs is small, as shown in Figure 1 and Table 2. There was less trapped water in the HGM3 specimen due to the larger wall thickness of HGM3 because the higher crushing strength is determined by larger wall thickness [30]. Therefore, the damping ratio of the HGM3 specimen was still lower than HGM1 and HGM2 specimens at the 50% replacement ratio level because the trapped air in HGM particles was dominant for the damping ratio improvement. In other words, the larger size HGMs contained more air in hollow structures, which more significantly affected the energy dissipation than the trapped water. Also, it can be found that the third peaks of HGM1, HGM2, and HGM3 specimens were very close to the RF specimen, which indicated that the three types of HGMs were non-reactive in the cement matrix.

**Figure 12:**
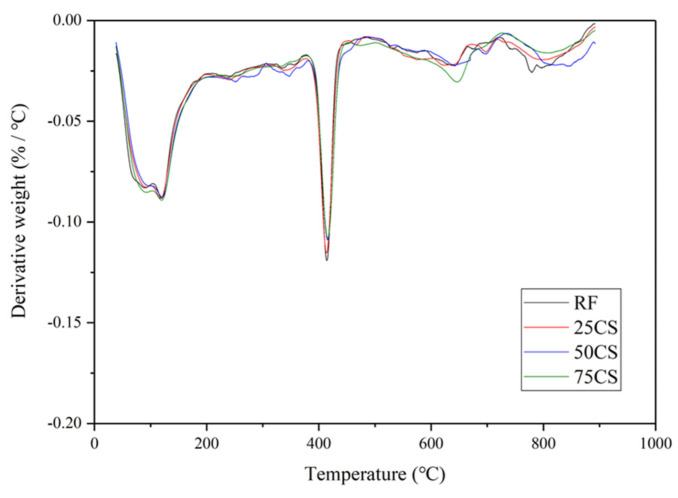
TGA results of CS specimens.

However, the TGA results of CS specimens did not have the same trend as HGM1 specimens. The first peak values of CS specimens were almost the same as the RF specimen when increasing the replacement ratio of CS, which means that there was no water trapped in the CS particle increased when CS content increased, as shown in Figure 12. Besides, compared to the HGM1 specimens, the first peak was lower at the same replacement ratio because there was no trapped water in the CS specimens due to the outer shell of the CS particle, which blocked the water from penetrating into the hollow structure. Therefore, at the replacement ratio of CS reached 75%, the increment of damping ratio was 9.80% compared to 25CS, which was three times that of HGM1 specimens. Besides, the second and third peaks of CS specimens were almost the same as the RF specimen, which indicated CS did not participate in hydration and pozzolanic reaction, so the compressive strength declined while increasing the CS content.

## 4. Conclusions

Three different types of microparticles were applied to develop vibration-reducible mortar specimens. The mechanical performance and damping properties were evaluated by compressive and flexural strength tests, damping ratio tests. Also, TGA analysis was performed to find if there is any reaction of HGMs and CS in mortar, which can affect the damping capacity. The important results of this study are summarized below:

(1) HGMs increased the damping ratio of mortar, although it decreased the compressive and flexural strength, especially when the crushing strength of HGM particles is low. However, the damping ratio can be increased by 10% with only a 4.1 MPa compressive strength decrement at a 25% replacement ratio of HGM1. The size of HGM is also an important factor, HGM1 provided higher damping ratio improvement than HMG2 and HGM3 due to its larger particle size. 

(2) There is no combination effect of HGM and other materials, whereas the application of AP, when incorporated with HGM, results in a drastic compressive strength decrease even with increasing damping ratio when incorporated with HGM. 

(3) CS also increased the damping ratio while decreasing the compressive and flexural strength due to weak ITZs between the cement matrix and its outer shell, although it had higher crushing strength than HGMs. However, the weak ITZs provide a sliding effect during vibration. Besides, there was no trapped water in the CS specimens revealed from TGA test results, and its size was much larger than HGM, so it can provide a better damping ratio improvement than HGM

(4) GFs improved the damping ratio as well as the flexural strength while decreasing the compressive strength. The compressive strength decrease was caused by flake shape of GFs. The folder on the GFs also provided the internal friction. The flexural strength increased because GFs worked as reinforcement. The SGFs were more effective than BGFs in damping capacity because SGFs have more surface area to be bonded with cement matrix. 

In summary, it can be found that all additives used in this study can improve the damping capacity while adversely affecting the compressive strength of specimens. However, CS and SGF are more effective than HGM. CS led to a 6.4 MPa compressive strength decrement but a 15% damping ratio increment at a 50% replacement ratio of CS. SGF can improve flexural strength while increasing the damping ratio by 20.83% with only a 10% replacement ratio.

The damping ratio results of cement mortar with various additives provide valuable information regarding the development of vibration-reducible cementitious material. Specifically, the microparticles containing voids inside can be promising for vibration reduction application. The limitation of the study is that it investigated the effect of microparticles on the damping ratio of mortar on a material scale (referred to as a lab-scale). For practical application, the studies on a structure scale (referred to as a real-scale) should proceed in future research. Besides, numerical studies should be conducted to simulate the structural damping considering interfacial friction between microparticles and cement paste.

## Figures and Tables

**Table 1:** Chemical composition of raw materials (mass%).

Material	SiO_2_	Al_2_O_3_	CaO	Fe_2_O_3_	TiO_2_	MgO	SO_3_	P_2_O_5_	Na_2_O
Cement	19.38	4.48	64.48	3.46	0.29	2.82	3.31	-	-
HGM	80.59	0.15	13.54	0.05	0.08	0.21	0.23	0.80	4.47
Cenosphere	67.28	23.22	0.31	1.51	0.39	0.38	-	0.49	0.47

**Table 2:** Physical properties of additives.

Materials	Median Particle Size (μm)	Density (kg/m^3^)	Crushing Strength (MPa)	Manufacturer
HGM1 [31]	55	150	2.1	3M^TM^
HGM2 [31]	45	460	41	3M^TM^
HGM3 [32]	16	600	193.1	3M^TM^
CS	407	650	70–140 [33]	Seokyung Cmt
Rubber particle (RP)	65	1450	-	Hangil RMB
Aluminum powder (AP)	-	2700	-	KMB Co., Ltd.
Natural fiber (NF) *	-	1797	-	Soo Industry Co.
SGF	81.5	2214	-	Merck KGaA
BGF	430	2260	-	Merck KGaA

* Natural fiber length: 10 mm.

**Table 3:** Mix proportions for specimens (kg/m^3^).

Mixture ID		Cement	Sand	HGM	RP	AP	NF	GF	CS	Water
RF *	867.0		867.0							390.2
25HGM1			650.3	12.5						390.2
50HGM1			433.5	25						390.2
50HGM2			433.5	76.7						390.2
50HGM3			433.5	100.0						390.2
75HGM1			216.8	37.5						390.2
30R			606.8		144.8					390.2
0.05AP			867.0			0.434				390.2
2NF			849.5				12.7			390.2
30R25HGM1			390.1	12.5	144.8					390.2
0.05AP25HGM1			650.9	12.5		0.434				390.2
2NF25HGM1			632.7	12.5			12.7			390.2
5SGF			823.0					37.6		390.2
5BGF			823.0					36.9		390.2
10SGF			779.0					75.3		390.2
10BGF			779.0					73.9		390.2
25CS			650.3						54.3	390.2
50CS			433.5						108.5	390.2
75CS			216.8						162.6	390.2

* RF: reference specimen, NF: natural fiber, RP: rubber particle, AP: aluminum powder, SGF: small size graphite flakes, BGF: big size graphite flakes.

## Data Availability

The data presented in this study are available on request from the corresponding authors.
